# The role of immune disfunction in schizophrenia pathogenesis

**DOI:** 10.1192/j.eurpsy.2023.2251

**Published:** 2023-07-19

**Authors:** I. Azevedo Silva, B. Martins, V. Melo, J. Cardão, A. Matos, C. Agostinho

**Affiliations:** 1 Psychiatry and Mental Health, Unidade Local de Saúde do Norte Alentejano, Portalegre; 2 Psychiatry and Mental Health, Centro Hospitalar do Médio Tejo, Tomar, Portugal

## Abstract

**Introduction:**

In the last years there has been increasing evidence that inflammation and autoimmunity may play a role in the pathogenesis of schizophrenia.

Although the brain has been considered an immune-privileged site, we understand now that infections and inflammation interfere with the blood-brain barrier, making the brain vulnerable to antibodies, cytokines and infectious agents.

**Objectives:**

To understand the role of immune disfunction in schizophrenia pathogenesis, as well as the potential role of immunotherapy in its treatment.

**Methods:**

We performed a narrative review of the evidence, using the following terms and their combinations “schizophrenia”, “autoimmunity” and “monoclonal antibodies”.

**Results:**

It is widely known that prenatal, perinatal and childhood exposure to infections, nutritional deficits and other environmental insults, acting on a background of genetic vulnerability, may lead to schizophrenia. In such cases, we can observe potent and enduring inflammatory responses, such as cytokines dysregulations.

State markers, including IL-1β, IL-6 and TGF-β have increased levels during exacerbation of symptoms and stabilized levels when antipsychotics are administrated. Trait markers, such as IL-12, IFN-γ and TNF-α have systematically increased levels in acutely and chronically ill patients, even during clinical stability.

Moreover, patients with schizophrenia have been showing abnormalities of the blood-brain barrier, signs of central nervous system inflammation and elevated autoantibody levels and reactivity.

Several autoimmune diseases are associated with schizophrenia, such as celiac disease, Graves’ disease and psoriasis. On the other hand, it is known since the 1950’s that schizophrenia has a negative association with rheumatoid arthritis.

There are case reports of people with psychosis that were treated with immunosuppressive agents (for concurrent autoimmune diseases) that showed improvement in their psychotic symptoms.

NSAIDs, immunomodulators and several monoclonal antibodies have been tested as potential treatments for schizophrenia. The results were conflicting but promising. It is suggested that not every patient with schizophrenia may benefit from these treatments. Ideally, treatment targeting the immune system should be provided in earlier phases of disease, such as in prodromal psychosis and first episode psychosis, because these are related to irreversible grey matter loss which causes cognitive decline.

**Conclusions:**

Immune dysregulation may have an important etiological role in schizophrenia.

Hence, specific therapeutic approaches targeting the immune system may lead to new ways of treating and even preventing psychotic disorders.

Further investigation is necessary in order to provide more information on how aberrant antibody and cytokine production interferes with neuronal function and how it is expressed at the clinical level.

**Disclosure of Interest:**

None Declared

